# Mechanical response of cardiac microtissues to acute localized injury

**DOI:** 10.1152/ajpheart.00305.2022

**Published:** 2022-09-02

**Authors:** Shoshana L. Das, Bryan P. Sutherland, Emma Lejeune, Jeroen Eyckmans, Christopher S. Chen

**Affiliations:** ^1^Harvard-MIT Program in Health Sciences and Technology, Institute for Medical Engineering and Science, Massachusetts Institute of Technology, Cambridge, Massachusetts; ^2^Department of Biomedical Engineering, Boston University, Boston, Massachusetts; ^3^Wyss Institute for Biologically Inspired Engineering, Harvard University, Boston, Massachusetts; ^4^Department of Mechanical Engineering, Boston University, Boston, Massachusetts

**Keywords:** cardiac fibrosis, cardiac mechanics, cardiac tissue engineering, iPSC-derived cardiomyocytes, organ-on-chip

## Abstract

After a myocardial infarction (MI), the heart undergoes changes including local remodeling that can lead to regional abnormalities in mechanical and electrical properties, ultimately increasing the risk of arrhythmias and heart failure. Although these responses have been successfully recapitulated in animal models of MI, local changes in tissue and cell-level mechanics caused by MI remain difficult to study in vivo. Here, we developed an in vitro cardiac microtissue (CMT) injury system that through acute focal injury recapitulates aspects of the regional responses seen following an MI. With a pulsed laser, cell death was induced in the center of the microtissue causing a loss of calcium signaling and a complete loss of contractile function in the injured region and resulting in a 39% reduction in the CMT’s overall force production. After 7 days, the injured area remained void of cardiomyocytes (CMs) and showed increased expression of vimentin and fibronectin, two markers for fibrotic remodeling. Interestingly, although the injured region showed minimal recovery, calcium amplitudes in uninjured regions returned to levels comparable with control. Furthermore, overall force production returned to preinjury levels despite the lack of contractile function in the injured region. Instead, uninjured regions exhibited elevated contractile function, compensating for the loss of function in the injured region, drawing parallels to changes in tissue-level mechanics seen in vivo. Overall, this work presents a new in vitro model to study cardiac tissue remodeling and electromechanical changes after injury.

**NEW & NOTEWORTHY** We report an in vitro cardiac injury model that uses a high-powered laser to induce regional cell death and a focal fibrotic response within a human-engineered cardiac microtissue. The model captures the effects of acute injury on tissue response, remodeling, and electromechanical recovery in both the damaged region and surrounding healthy tissue, modeling similar changes to contractile function observed in vivo following myocardial infarction.

## INTRODUCTION

Each year, over one million people in the United States experience a myocardial infarction (MI). After an MI, the infarcted tissue undergoes a focal fibrotic response leading to tissue heterogeneity across the myocardium, a fibrotic-scarred region surrounded by healthy myocardium (1–4). Although the presence of this scar is important for redistributing wall stresses within the heart and preventing rupture (2, 5, 6), the excessive extracellular matrix (ECM) and lack of cardiomyocytes (CMs) within the scar yield altered regional mechanical and electrical properties, affecting the function of the surrounding myocardium and overall heart function (6, 7).

After an infarct, collagen degradation leads to the process of infarct expansion and creates increased wall stresses at the infarct border. This stretching results in the cross-sectional thinning (and areal expansion) of the infarct region and contributes to overall ventricular dysfunction and dilation (8–10). Over time, the response to local damage leads to thinning of the myocardium and the deposition of a collagen scar, ultimately decreasing contractile activity (8, 11, 12). The surrounding uninjured regions augment their contraction because of the increased local stretch, resulting from the increased preload because of hypokinesis of the infarct zone, leading to a compensatory boost in contraction at the expense of causing increased metabolic stress in the peri-infarct regions (13–15). Over time, the increased workload and stress cause adverse remodeling of the myocardium, contributing to heart failure in patients (7, 16–18).

Understanding the complexities of cardiac remodeling after MI and how this remodeling leads to heart failure has been challenging. The consequences of MI on the myocardium and cardiac function have been traditionally investigated in animal models; however, studying the dynamics of cell and tissue level remodeling are challenging to observe in vivo. The emergence and advancement of engineered heart tissues generated from human-induced pluripotent stem cell (iPSC)-derived cardiomyocytes in the last decade provide an alternative approach to better understand cardiac biology and pathophysiology at this cellular and tissue level. The in vitro nature of these models enables the study of human cells and the ability to investigate both mechanical and electrical properties of heart tissue all within controlled and manipulable environments (19–24). Specifically, the use of engineered tissues and iPSC technologies provides certain advantages over other in vitro models like tissues slices, as they enable the study of specific cell populations in isolation, the use of gene-editing tools, and the use of patient-specific cells. Although strategies to model MI have been achieved by applying hypoxia to the entire tissue (22, 23, 25, 26), altering cell populations (27, 28), or inducing fibrotic responses by the addition of external factors such as transforming growth factor (TGF)-β1 (29–33), current models capture neither the acute, localized response to the cell death nor how this damage affects the surrounding tissue, as occurs during an MI.

Here, we present a model for myocardial injury, which uses a high-power pulsed laser to regionally damage a cardiac microtissue (CMT), to mimic the regional loss of cells after an infarct. This results in an injured region within the center of the tissue where the surrounding myocardium maintains function and over time augments it, enabling the study of the effect of localized acute cell death, its progression into fibrosis, and the effect this has on the surrounding tissue.

## MATERIAL AND METHODS

### Cell Sources

A previously reported human iPSC line derived from the Personal Genome Project (PGP1; 55-yr-old male) containing fluorescently tagged green fluorescent protein (GFP) tagged titin (TTN), a *Z*-disk protein, (TTN-GFP) was used for these studies (34). Adult human ventricular cardiac fibroblasts (CFs, Cell Applications) were from a 26-yr-old male.

### Cell Preparation and Culture

iPSCs were maintained in a complete mTeSR1 medium (Stem Cell) and differentiated into monolayers following small molecule protocol (35). Briefly, cells were differentiated using RPMI 1640 medium, GlutaMAX (Gibco) supplemented with B-27 supplement, minus insulin (Gibco). CHIR90021 (12 μM; Tocris) was added for 24 h on *day 0* of differentiation and then 5 μM IWP4 (Tocris) was added for *days 2* and *3*. On *day 9*, cells were switched to RPMI 1640 medium, GlutaMAX containing standard B-27 supplement (Gibco). After 2 days, CMs were metabolically selected using glucose-free RPMI 1640 medium (Gibco) supplemented with 4 mM of DL-lactate (Sigma) for 4 days. Following selection, CMs were replated onto fibronectin (10 μg/mL; Corning)-coated plates and maintained in RPMI 1640, supplemented with B-27. CMs were used within 7 days of replating. CFs were maintained in human cardiac fibroblast growth medium (Cell Applications) and used at *passage 4*.

### Microtissue Seeding

Polydimethylsiloxane (PDMS) microtissue devices with wells containing two cylindrical micropillars with spherical caps were cast from three-dimensional (3-D) printed molds (Protolabs). Two days before seeding, devices were plasma treated for 60 s, treated for 2 h with 0.01% poly-l-lysine (ScienCell), and then for 15 min with 0.1% glutaraldehyde (Electron Microscopy Sciences). PDMS devices were then washed and soaked in deionized water at 4°C until use. Before seeding, devices were briefly soaked in 70% ethanol, dried, then sterilized under ultraviolet for 15 min. To prevent cell attachment to the bottom of wells, wells were treated with 2% Pluronic F-127 (Sigma) for 30 min and dried with compressed air.

To make each tissue, 60,000 cells (90% iPSC-CMs and 10% human CFs) were suspended in 7.5 μL of an ECM solution consisting of human fibrinogen (4 mg/mL; Sigma-Aldrich), 10% Matrigel (Corning), 0.4 U of thrombin (Sigma-Aldrich) per milligram of fibrinogen, 5 μM Y-27632 (Tocris), and aprotinin (0.033 mg/mL; Sigma-Aldrich) were added to each well. After allowing for gel polymerization for 10 min, tissue maintenance medium containing high-glucose Dulbecco’s modified Eagle’s medium (Thermo Fisher Scientific) supplemented with 10% fetal bovine serum (Sigma-Aldrich), 1% penicillin-streptomycin (Thermo Fisher Scientific), 1% nonessential amino acids (Thermo Fisher Scientific), 1% GlutaMAX (Thermo Fisher Scientific), 5 μM Y-27632, and 0.033 mg/mL aprotinin were added. After 2 days, Y-27632 was removed, and after 7 days, the aprotinin concentration was decreased to 0.016 mg/mL.

### Laser Injury

After 7 days of tissue formation, tissues were damaged using a 1,064-nm Pulsed Nd:YAG Laser (Minilite, Continuum). Twelve pulses at 1 mJ were delivered in a 3 × 4 grid pattern to the center region of each microtissue.

### Cell Death Staining, Immunofluorescence Staining, and Confocal Microscopy

For cell death staining, 1 h after laser injury, microtissues were stained with Apoptosis/Necrosis Assay Kit (Abcam) following the manufacturer’s protocol. Samples were then fixed in 4% paraformaldehyde for 20 min and washed three times in PBS. Before imaging, samples were stained with 1:1,000 DAPI for 1 h at room temperature (RT). For antibody staining, samples were first fixed in 4% paraformaldehyde for 20 min, washed three times in PBS, and then blocked for 1 h at RT in 2% BSA in PBS. Then anti-vimentin antibody (Abcam, ab8978, RRID:AB_306907) at a 1:1,000 dilution and anti-fibronectin (Abcam, ab32419, RRID:AB_732379) at a 1:150 dilution in 2% BSA in PBS were added to samples, and samples were incubated overnight at 4°C, followed by three washes in PBS. Secondary antibodies (Invitrogen, ThermoFisher, A32795, RRID:AB_2866496, A11030, RRID:AB 2534089) and DAPI at 1:1,000 dilutions were then added and incubated overnight at 4°C, followed by three washes in PBS before imaging.

*Z*-stacks of samples were imaged on a Leica SP8 confocal microscope with a Leica ×25 water objective and Leica LAS X imaging software. To determine the ratio of necrosis to apoptosis for injury samples, the threshold function in ImageJ was used to create masks for necrosis and apoptosis. The apoptosis mask was subtracted from the necrosis mask to remove any cells at later stages of apoptosis, and the positive area of this mask (necrosis) was compared with that of the apoptosis mask for each sample. For DAPI analysis, 150- by 250-μm regions in the center of the tissue were taken of the maximum projections of acquired stacks, and the median intensity value was measured as a proxy for cell number. For vimentin and fibronectin analysis, maximum projections of acquired stacks were taken, and the mean signal intensity was measured and reported.

### Sarcomere Analysis

In this code, we segmented “myofibril-like” structures from fluorescent *z*-stack images (sarcomere analysis and strain analysis codes may be found at https://github.com/elejeune11/Das-manuscript-2022). A 150- × 250-μm region of interest in the center of stacks was selected to represent the injury region. For each slice, Laplacian filtering was applied to detect the steep gradient in brightness found at the edges of the myofibrils (36). A multidimensional Gaussian filter with a standard deviation of two was then applied to the resulting Laplacian filtered image (37), followed by the application of an Otsu threshold (38). Speckle noise (present in a small subset of images) was removed by multiplying the resulting binary image by a binary mask created by applying an Otsu threshold to the original image after a median filter with size three is applied (37). The resulting segmented region in the binary image was identified using the scikit-image “regionprops” function (38). To reduce noise and select for fibril structures, minimum segment length of 6 μm was implemented, and all segmented regions with a major axis length longer than 6 μm were saved and the total length summed.

### Mechanical Property Characterization

The mechanical properties of the tissues were characterized using nanoindentation (Piuma, Optics 11) equipped with a probe (Piuma) possessing a 55-μm bead diameter and a spring constant of 0.18 N m^−1^. The Young’s Modulus was averaged from at least four indentations at the center of each tissue that were averaged for each individual sample (*n* = 2, 3, or 4) for each of four experimental repeats. Curves were fitted to a Hertz contact model fitted to the loading curve assuming a Poisson ratio of 0.5. All measurements were taken with tissues submerged in tissue maintenance medium supplemented with 70 mM potassium chloride (Fisher) and performed on *day 0* and on *day 7* postinjury.

### Calcium Imaging

Immediately after injury (*day 0*) and on *day 7* after injury, Rhod-3 Calcium Imaging Kit (Invitrogen) was applied to injured and control tissues according to the manufacturer’s protocol. Briefly, microtissues were incubated with dye solution at RT for 45 min, washed, and incubated another 45 min at RT to allow for ester cleavage. Samples were imaged in Tyrode’s salts with sodium bicarbonate (Sigma). Time-lapse videos of the microtissue’s spontaneous contractions were acquired at either 30 or 15 frames per second on a Nikon Eclipse Ti with a ×4 objective with an Evolve EMCCD camera in a humidified chamber at 37°C and 5% CO_2_. Regions of interest (ROIs) for the center region (299 μm × 199 μm) and edge region (100 μm × 100 μm) of microtissues selected and the change in intensity over time were measured. A two-frame moving average was taken to reduce noise. The amplitude of the calcium waveform was then calculated. As Rhod-3 is not a ratiometric dye, we normalized to the average amplitude for the center ROI of the control for each imaging session for better comparison between experimental replicates.

### Force Measurements

Time-lapse videos of the microtissue’s spontaneous contractions were taken before laser injury, 30 min after laser injury, and then subsequently on *days 1*, *3*, *5*, and *7* postinjury to quantify changes in force over time. Videos were acquired at 30 frames/second on a Nikon Eclipse Ti (Nixon Instruments) with a ×4 objective with an Evolve EMCCD Camera (Photometrics). The microscope was equipped with a temperature and CO_2_-equilibrated environmental chamber. Maximum contractile force, stress, and contraction kinetics were calculated using a custom Matlab (RRID:SCR_001622). Script based on the deflection of the pillars and the measured pillar spring constant of 2.68 μN/μm, as previously described (39). Each tissue was then normalized to its *day 0*, before injury twitch force.

### Strain Analysis

In this code, we compute the average strain in manually defined regions of interest (ROIs) in time-lapse videos of microtissues (code can be at https://github.com/elejeune11/Das-manuscript-2022). A center region ROI (299 μm × 199 μm) and an adjacent region ROI (139 μm × 299 μm) were selected for each tissue. Following image scaling (38, 40), Shi-Tomasi corner points in both ROIs were detected (36, 41) and tracked using the iterative Lucas–Kanade method with pyramids (36, 42). The absolute displacement of all corner points in each frame was computed, and the absolute displacement peaks were detected with the SciPy “signal.find_peaks” algorithm (37). Temporal reference points were defined as all points halfway between each peak. For *n* peaks, there were *n*-1 temporal reference points and thus *n*-2 temporal periods per time-lapse video. The strains were then computed for both the center region ROI and adjacent region ROI with respect to the initial frame of each temporal reference period. For each temporal reference period, the center and edge ROIs were treated separately and the Shi-Tomasi corner points were recomputed, and tracking was performed for the duration of the temporal period.

For each ROI and temporal period, the change in position of each corner point with respect to the initial frame was used to compute the average deformation gradient of the ROI with respect to frame number (43). The average deformation gradient was then transformed into the Green–Lagrange strain tensor and the maximum strain in the *xx* direction (defined by the axis connecting the two microtissue posts, maximum refers to maximum magnitude as contractile strain is negative) was computed. For each timelapse image, the final output is then the maximum contractile strain in the *xx* direction (*E_xx_*) averaged over all temporal periods detected in the timelapse for the center and edge ROIs, respectively.

### Statistical Analysis

Graphpad Prism 9 (RRID:SCR_000306) was used to perform all statistical analysis. A two-way ANOVA statistical test was used to determine significance for multiple repeat experiments across conditions with the exceptions of Fig. 2, *B* and *C* and Fig. 4, *D* and *F*, where a Student’s *t* test was used. Significant values were determined at *P* < 0.05, *P* < 0.01, *P* < 0.001, and *P* < 0.0001.

## RESULTS

### Focal Laser Injury to Engineered Cardiac Tissues Leads to Regional Cell Death

**Figure 1.**
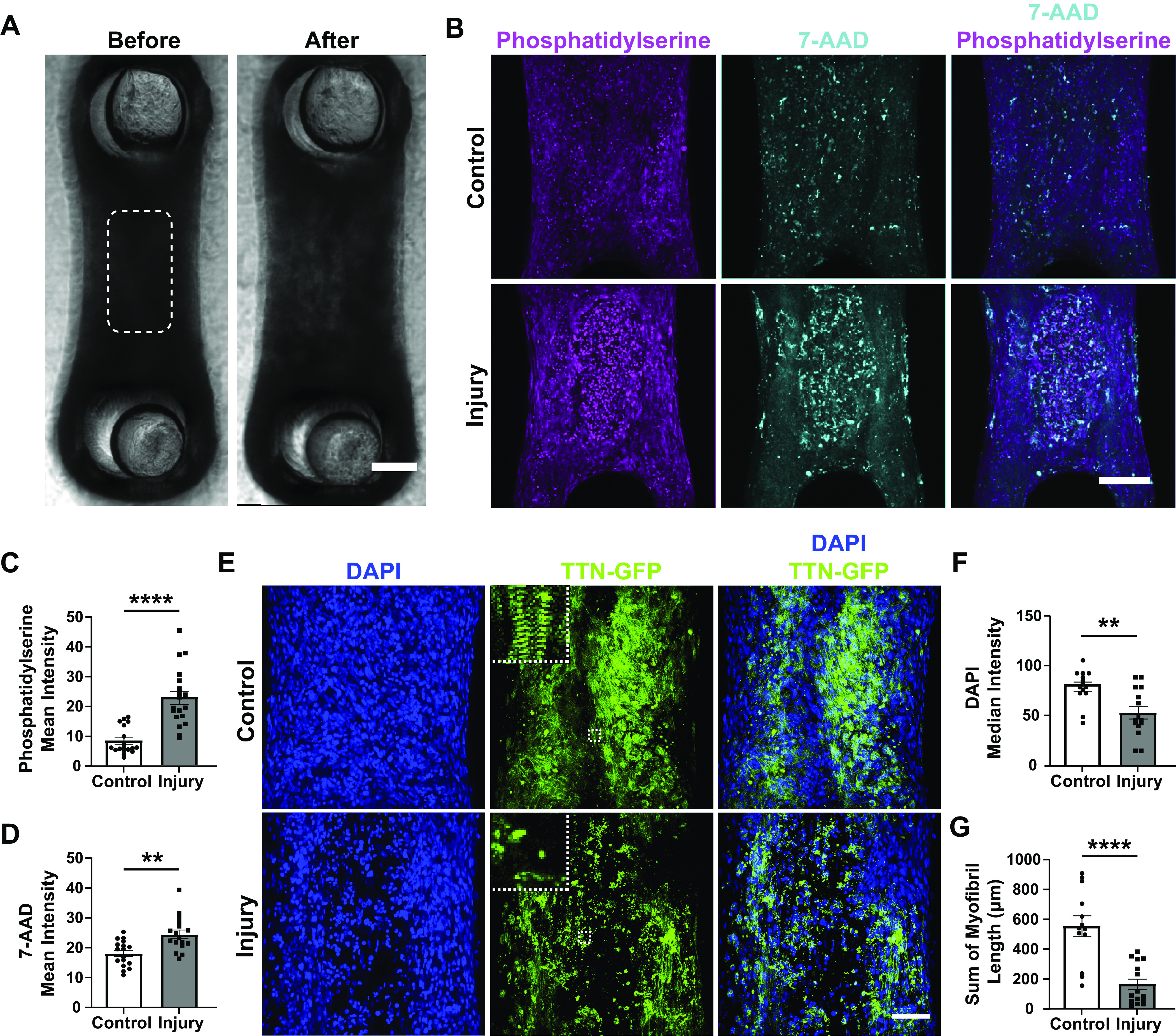
Focal laser injury to engineered cardiac tissues leads to regional cell death. *A*: brightfield images of CMTs 7 days postseeding before (*left*) and after (*right*) laser ablation. White dashed box indicates region of targeted laser injury. Scale bar = 200 μm. *B*: maximum projections of fluorescence images for phosphatidylserine (apoptosis, magenta) and 7-aminoactinomycin D (7-AAD) (necrosis, cyan) of control (*top*) and injury (*bottom*) conditions 2 h postinjury. Scale bar = 200 μm. *C*: mean intensity of phosphatidylserine of *z*-stacks in center region of tissues. ****P < 0.0001; *n* = 18 (control) and 19 (injury). *D*: mean intensity of 7-AAD of *z*-stacks in center region of tissues. **P < 0.01; *n* = 18 (control) and 19 (injury). *E*: maximum projections of fluorescence imaging of DAPI (blue) and TTN-GFP (green) of control (*top*) and injury (*bottom*) tissues 2 h after injury. *Insets*: white dashed boxes are 25 μm × 25 μm. Scale bar = 100 μm. *F*: median intensity of DAPI of *z*-stacks in center region of tissues. **P < 0.01; *n* = 14 (control) and 15 (injury). *G*: average of sum of myofibril length over slices from *z*-stacks of center region of tissues. ****P < 0.0001; *n* = 13 (control) and 15 (injury). Bar plots represent means ± SE. CMT, cardiac microtissue; GFP, green fluorescent protein.

An MI causes cell death to a region of the heart that triggers a fibrotic response and results in a region of tissue that has a different cellular composition and different mechanical and electrical properties compared with the rest of the myocardium (2, 6, 44–47). To capture this phenomenon, we sought to develop a platform, which could target injury to a region of a CMT to study the effect of an insult on the region and on adjacent tissue. Since laser light is highly tunable and has a high degree of spatial control, we sought to use a laser to deliver highly targeted regional cell damage to CMTs. To do this, CMTs were seeded from a combination of human-induced pluripotent stem cell (iPSC)-derived CMs with GFP-tagged titin and primary human CFs. After allowing the tissue to form for 7 days, CMTs were then damaged to induce cell death in the center of the CMT using a Nd:YAG nanosecond-pulsed laser (Fig. 1*A*).

To avoid tissue ablation, the energy of the laser pulse was set to 1.0 mJ to cause cell death without creating a full-thickness hole in the microtissue. The laser pulses were targeted to the center region of the tissue to create a focal injury (Fig. 1*A*). After an MI, cells die from a combination of apoptotic and necrotic processes (45–47). To characterize the cell death caused by the laser, tissue samples were fixed 2 h after injury and stained for phosphatidylserine and 7-aminoactinomycin D (7-AAD), markers of apoptosis and necrosis, respectively (Fig. 1*B*). Both markers stained with significantly higher intensity in the region of injury compared with uninjured CMTs (Fig. 1, *C* and *D*), and the relative ratio of necrosis to apoptosis was 2:1. Furthermore, nuclear staining with DAPI revealed significant cell loss in the injured region (Fig. 1, *E* and *F*). Closer examination of GFP-tagged sarcomeres showed a dramatic loss of myofibrils in injured compared with noninjured tissues (Fig. 1, *E* and *G*). Overall, these data show that a pulsed laser can be used to regionally induce cell death via both apoptosis and necrosis within a CMT.

### Injured Tissues Demonstrate Increased ECM Production over Time

**Figure 2.**
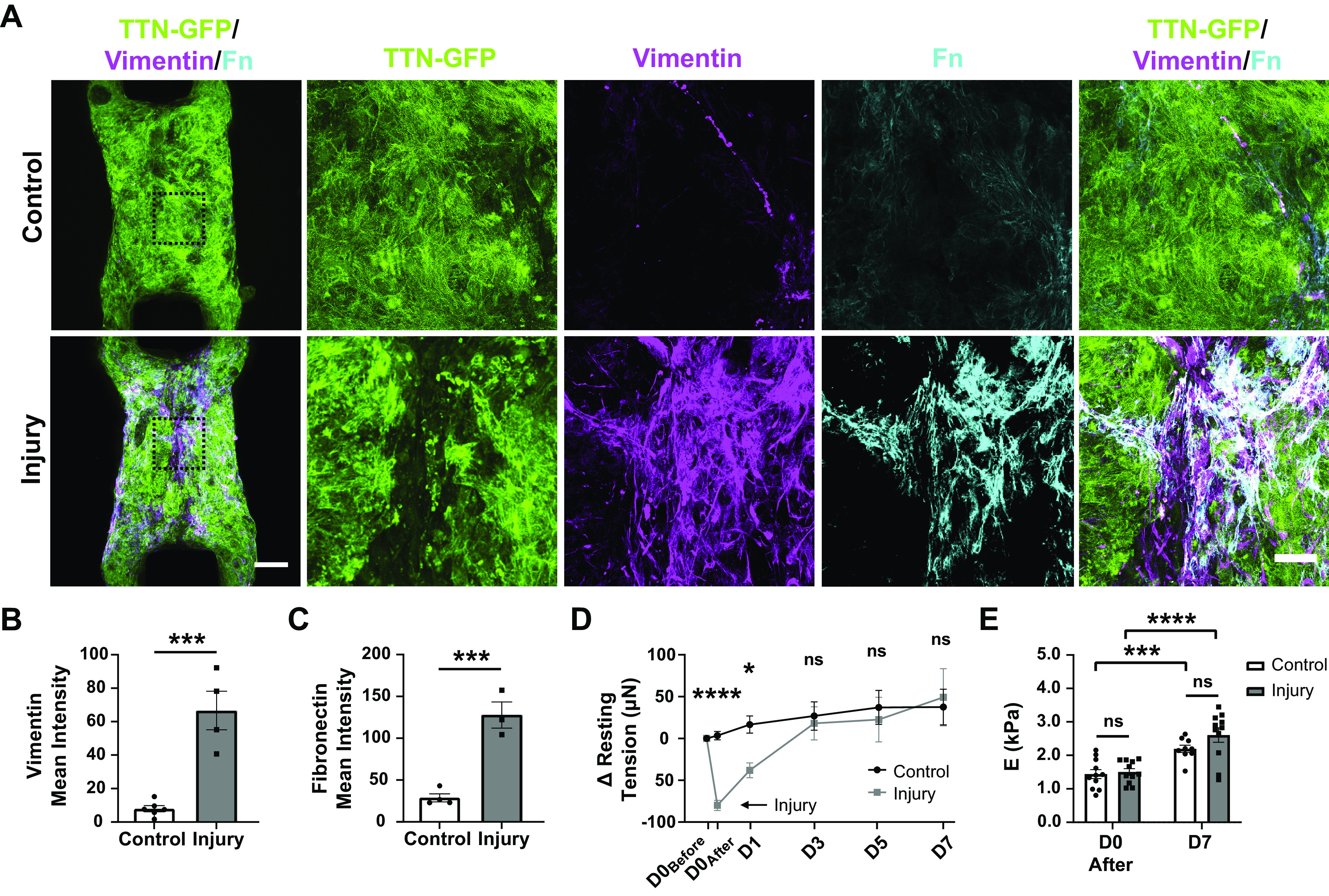
Injured tissues demonstrate increased ECM production over time. *A*: maximum projections of CMT on *day 7* postinjury with TTN-GFP (green), vimentin (magenta), and fibronectin (cyan); low-magnification (*left*), scale bar = 200 μm, black dashed boxes indicate corresponding high-magnification images (*right*), scale bar = 50 μm. *B*: mean intensity of vimentin for sum of *z*-stacks in center region of tissues. ***P < 0.001; *n* = 6 (injury) and 4 (control). Bar plots represent means ± SD. *C*: mean intensity of fibronectin for sum of *z*-stacks in center region of tissues. ***P < 0.001; *n* = 4 (injury) and 3 (control). *D*: change in resting tension from before injury over time for control (black) and injured (gray) tissues. ****P < 0.0001, *P < 0.05, *n* = 26 (injury) and 25 (control). *E*: Young’s modulus measured in the center region of the tissue at *day 0* after injury and *day 7* after injury. ***P < 0.001, ****P < 0.0001; *n* = 9–11. Bar plots represent means ± SE. Brackets above bars indicate significance or nonsignificance in indicated pairwise comparisons. CMT, cardiac microtissue; ECM, extracellular matrix; GFP, green fluorescent protein.

Upon establishing the injury model, we next sought to characterize the response of CMTs to injury. After a hypoxic event to the heart, myocardium undergoes inflammatory and fibrotic processes that lead to an increase in fibroblasts, which then deposit a fibronectin and collagen type I-rich scar tissue (1, 2, 44, 48). To investigate if our model captures this fibrotic tissue response, CMTs were fixed 7 days after injury and stained for vimentin and fibronectin to assess fibroblast activation and fibrotic remodeling. The injured region showed a low TTN-GFP signal suggesting that uninjured CMs did not migrate into the injured area. Instead, expression of vimentin (Fig. 2, *A* and *B*) and fibronectin (Fig. 2, *A* and *C*) was highly upregulated in the injured region, suggesting that the laser injury resulted in an activation of fibroblasts, which then deposited a fibronectin-rich ECM that is reminiscent of replacement fibrosis found post-MI in vivo. As fibroblasts also contribute to the resting, baseline tension within the tissues, we measured the change in resting tension caused by the injury and how it changed over time (Fig. 2*D*). Injury led to a −80 ± 6 μN (mean ± SE) change in resting tension, compared with the control (3 ± 5 μN; mean ± SE). By *day 3*, the resting tension in the injured tissues recovered to similar levels as control tissues and continued to trend with the control tissues through *day 7*. These data support that injury impacts both diastolic and systolic changes in this model.

A hallmark of scar tissue post-MI is an increase in stiffness of the remodeled ECM (49–51). To assess if the remodeling of the injured region affected the mechanical properties of the CMT, we measured the stiffness (Young’s modulus) in the injured region of the tissue using nanoindentation. To ensure the laser injury did not affect the mechanical property of the tissue, tissue stiffness at the site of injury was measured immediately after the injury (*day 0*), and 7 days later (Fig. 2*E*). Immediately after injury, control and injured tissues had similar stiffnesses of 1.4 ± 0.1 kPa (mean ± SE) and 1.5 ± 0.1 kPa (mean ± SE), respectively. After 7 days, the tissue stiffness was increased in both conditions, and although the stiffness between injured (2.6 ± 0.2 kPa) and control tissues (2.2 ± 0.1 kPa) was not significantly different, the stiffness in the injured CMTs did trend higher (*P* = 0.0795). Together, our data show that laser injury can induce remodeling processes of replacement fibrosis in a CMT.

### Electrical Function is Regained in Adjacent Tissue but Not in the Region of Injury

**Figure 3.**
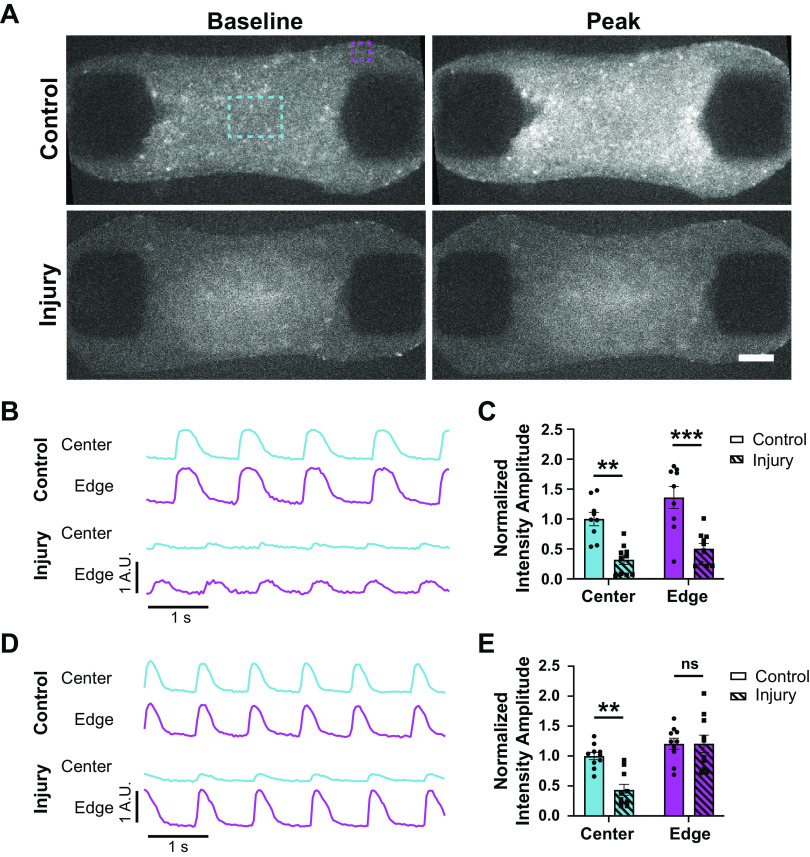
Electrical function is regained in adjacent tissue but not in the region of injury. *A*: widefield fluorescence images of control (*top*) and injured (*bottom*) CMTs stained with Rhod-3 calcium dye 2 h postinjury showing baseline (*left*) and peak (*right*) signal. Dashed boxes indicate regions of interest for traces and analysis in *B–E*. Center region (cyan) and edge region (magenta). Scale bar = 200 μm. *B*: example traces for a control and an injury tissue at *day 0* after injury in center (cyan) and edge (magenta) regions indicated in *A.* All traces are normalized to control center region trace. *C*: normalized intensity amplitudes for traces at *day 0* after injury. Normalized to control center region. **P < 0.01, ***P < 0.001; *n* = 9–10. *D*: example traces for a control and an injury tissue at *day 7* after injury in center (cyan) and edge (magenta) regions indicated in *A.* All traces are normalized to control center region trace. *E*: normalized intensity amplitudes for traces at *day 7* after injury. Normalized to control center region. **P < 0.01; *n* = 10. Bar plots represent means ± SE. CMT, cardiac microtissue.

Given that CMs are the primary conductors of electrical signals through the myocardium, the loss of CMs after an MI renders the affected region nonexcitable (52, 53). To evaluate if our model could capture this clinical feature of MI, Rhod-3 AM calcium dye was added to the medium of injured and control tissues, and the amplitudes of the calcium signal were measured in the region of injury and in the adjacent tissue (Fig. 3*A*, Supplemental Videos S1–S4; all Supplemental material is available at https://doi.org/10.6084/m9.figshare.20020511). After the injury, at *day 0*, the amplitude of the calcium wave was reduced in both the center and adjacent regions compared with the control tissue (Fig. 3, *B* and *C*). Although the amplitude in the center of injury, decreased by 69 ± 8% (mean ± SE) relative to the control, the adjacent region was reduced by only 49 ± 10% (mean ± SE). Thus, although the injury had the largest effect directly on the region of injury, the electrical activity was surprisingly still affected in neighboring regions.

To understand if this effect persisted long term, calcium amplitude was measured again in the same tissue regions at *day 7* postinjury. Although the amplitude in the control and in the center of the injury were both unchanged from the *day 0* measurements, the adjacent region of the injured condition regained calcium amplitudes like that of the control (Fig. 3, *D* and *E*). These data indicate that local injury may initially impair the electrical function of CMs, which are located outsides of the injured region, but over time these adjacent regions recover normal electrical function. In contrast, the electrical activity in the injured region fails to recover mimicking the poor electrical properties of a myocardial scar in vivo.

### Force Recovery over Time Corresponds with Increased Contraction in Regions Adjacent to Injury

**Figure 4.**
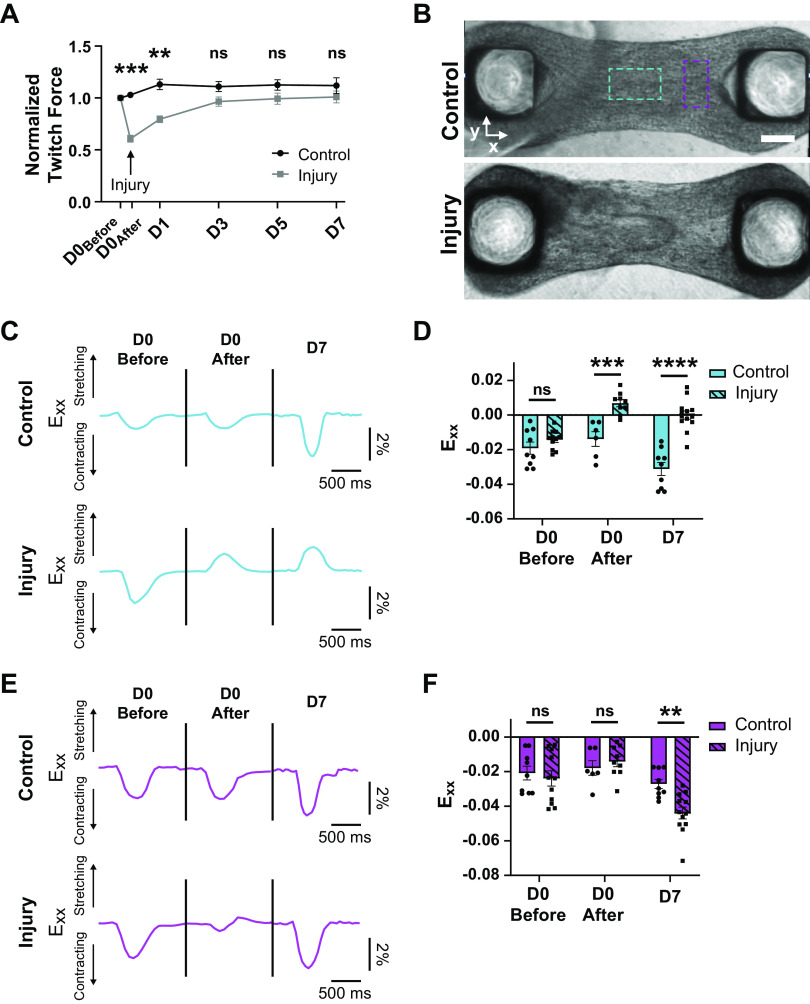
Force recovery over time corresponds with increased contraction in regions adjacent to injury. *A*: normalized twitch force before injury and over time after injury for control (black) and injured (gray) tissues normalized to before injury force for each tissue. ***P < 0.001, **P < 0.01; *n* = 26 (injury) and 25 (control). *B*: brightfield images of control (*top*) and injured (*bottom*) CMT postinjury. Dashed boxes indicate regions of interest for traces and analysis in *C–F*. Center region (cyan) and adjacent region (magenta). Scale bar = 200 μm. *C*: example traces of *E_xx_* for the same control (*top*) and injury (*bottom*) tissues over time in center region indicated in *B* (cyan) at *day 0* before injury, *day 0* after injury, and *day 7* after injury. *D*: *E_xx_* in center regions as defined in *B* (cyan) for control and injury tissues at *day 0* before injury, *day 0* after injury, and *day 7* after injury. **P < 0.01, ****P < 0.0001; *n* = 6–13. *E*: example traces of *E_xx_* for the same control (*top*) and injury (*bottom*) tissues over time in adjacent region indicated in *B* (magenta) at *day 0* before injury, *day 0* after injury, and *day 7* after injury. *F*: *E_xx_* in adjacent regions as defined in *B* (magenta) for control and injury tissues at *day 0* before injury, *day 0* after injury, and *day 7* after injury. *P < 0.05, *n* = 6–13. Bar plots represent means ± SE. CMT, cardiac microtissue.

The loss of CMs and subsequent remodeling after an MI also negatively impacts overall cardiac contractility (7, 51). To study the ramifications of injury on contractile function in our model system, we measured the deflection of the flexible pillars to which the microtissues were attached and calculated the twitch force generation (39; Supplemental Videos S5–S8). Laser injury reduced the twitch force by 39 ± 3% (mean ± SE) compared with the baseline (Fig. 4*A*). Twitch force for the same tissues was then tracked over the next 7 days (Supplemental Videos S9 and S10). Although in the control samples force generation remained close to baseline, the twitch force of the injured tissues increased over time after the initial drop, returning close to the baseline level for these tissues after several days (Fig. 4*A*). In two of the injury timepoints, immediately after injury and at *day 1* after injury, we observed asynchronous beating in 19% and 4% of tissues, respectively. This asynchrony could be relevant for the study of arrhythmogenesis in this model. To understand the contribution of this asynchrony on the observed reduced force production following injury, we excluded these tissues from the twitch force analysis and found that this exclusion did not alter the findings. We, therefore, conclude that the drop in force resulted from a loss of contractile function from injury.

In vivo studies of the mechanics of scar tissue in the heart show that not only does the scar region stop contracting, but it is also cyclically stretched by the surrounding contracting cardiac tissue, which negatively impacts overall pumping function (54). Since injured CMTs recapitulate a similar heterogeneity within the tissue, we more closely investigated the mechanics of the scar-like and adjacent tissue over time by measuring the strain in the direction of contraction *(E_xx_*) in the injured region and the adjacent tissue before injury, after injury, and at *day 7* postinjury (Fig. 4*B*). Before injury, the center region had average strains of −1.4 ± 0.2%, which was similar to the −1.9 ± 0.4% (mean ± SE) of the control. After injury, this region lost its contractile function and instead was being stretched by the surrounding contraction; this was indicated by a change to a positive strain of +0.6 ± 0.2% (mean ± SE), compared with the control which maintained a negative (i.e., contractile) strain of −1.4 ± 0.4% (Fig. 4, *C* and *D*). At *day 7*, this region no longer stretched during tissue contraction but instead was akinetic (+0.02 ± 0.4%; mean ± SE), indicating the injured region did not regain contractile function and therefore did not contribute to the overall twitch force recovery.

We next observed the effect of the injury on the mechanical function of the adjacent tissue. In contrast to the center region of the tissue, the injury did not reduce the contractile function of the adjacent region. Right after injury, there was a slight, but nonsignificant, reduction in the strain magnitude, but overall contractile function was maintained (Fig. 4, *E* and *F*). Interestingly, at *day 7*, the magnitude of *E_xx_* in the injured condition was twice the control at −5.5 ± 1.3% (mean ± SE) compared with −2.8 ± 0.4% (mean ± SE). These data suggest that the CMs in the adjacent regions increased contractile function over time, leading to a compensation for the lack of contraction in the injured region, which would explain the force recovery observed in Fig. 4*A*, suggesting that the functional myocardium adjacent to the scar is adapting in response to the scar-like region as a boundary condition. These findings present an interesting context to enable the study of border zone effects by the mechanics of cardiac scars in an in vitro setting.

## DISCUSSION

In this work, we demonstrate the creation of a focal cardiac injury and fibrosis model through a laser-induced, localized injury in a cardiac microtissue. By inducing injury via laser, the model captures the acute loss of function, the subsequent response to injury, and the compensatory recovery mechanisms at the injury site and in surrounding regions. These features provide a human in vitro model that captures aspects of the response and remodeling which occur after an infarct.

After an acute MI in vivo, the body and myocardium respond to adapt to the sudden loss of CMs. The body responds neurohormonally; the sympathetic adrenergic system is activated to increase heart rate and contractility to temporarily maintain cardiac output. Then locally, starting just hours after insult, the infarct zone undergoes infarct expansion, where degradation of ECM leads to wall thinning and dilation, causing increased wall stress (8–10). These local changes to loading engage Frank–Starling relationships and induce compensatory mechanisms in the surviving myocardium, leading to increased contraction of myocytes in the border zone and surrounding regions. However, despite this hyperkinesis, much of this effect is expended in the stretching of the infarct zone during cardiac contraction, a process known as systolic bulging (12–15). In the longer term, larger-scale remodeling occurs, including myocyte hypertrophy and changes in the ventricular architecture to better distribute the increased wall stresses (8, 11, 13). Over time these remodeling changes lead to progressive dilation, scar expansion, decreased function, and can ultimately lead to heart failure.

In our CMT injury model, we observe a similar loss of contraction in the injured region and even stretching of this region, potentially representing aspects of systolic bulging found in vivo (13, 15). Analyzing the strain in the injury-adjacent regions 7 days after injury, we found increased shortening during contraction, mirroring the hyperkinesis observed in the border zone and surrounding regions in in vivo infarcts (13, 14). Although this model captures a regional compensatory mechanism to acute CM loss, it does not have the response induced by the adrenergic system seen in vivo. The ability to study the response without this neurohormonal activation could provide an opportunity to isolate and better understand the loading and stress-driven responses post-acute injury and their effects on local CMs.

In vivo, after an acute hypoxic event, the regional replacement fibrosis is initiated by local cell death which triggers an extensive immune response characterized by the recruitment of neutrophils and macrophages to the hypoxic region, which clear dead cells and debris and activates a subsequent fibrotic response. Fibroblasts become activated and build the initial granulation tissue, which becomes the template for scar formation and eventual scar maturation (55–57).

The model we present here demonstrates some aspects of this in vivo response including local cell death, granulation tissue formation, and the start of scar formation; however, the model does not capture other important aspects of the postinfarction in vivo response. For example, as the system is only composed of CMs and CFs, it lacks immune cells to clear cell debris and mount a true inflammatory response. In our injury model, additional investigation is needed to characterize the fate of cell debris, and how the lack of immune cells affects subsequent processes. Interestingly, even in the absence of immune cells, the model still appears to capture signs of a fibrotic response as evidenced by notable increases in both vimentin and fibronectin. These observations suggest that the ability of fibroblasts to densify and replace areas experiencing CM loss may be sufficient to drive some of the compensatory changes in cardiac tissue following infarct. One potential mechanism for this process could be that the induced apoptotic and necrotic cell death in our model may elicit a nonimmune cell-mediated inflammatory response, which activates the fibroblasts to deposit ECM and increase vimentin expression (58–61), and warrants further investigation. In addition, although the model shows evidence of early fibrotic processes, it does not capture scar formation and maturation owing to the short duration of the current studies. Studies with extended duration and that incorporate more immune components could enable the extension of this model to study the more elaborate fibrotic responses observed in vivo. Lastly, the method of injury via laser, although achieving similar cell death observed in infarction, bypasses many important pathways induced by injury via hypoxia. Hypoxia leads to the activation of numerous stress-induced signaling pathways including but not limited to damage-associated molecular patterns, complement, and Toll-like receptor signal activation, inflammasome release, induction of C-reactive protein, all of which impact inflammatory and fibrotic responses (58, 59, 61). As such, the experimental platform introduced here should be considered an additional tool, rather than a replacement for, existing approaches to investigate injury responses.

Despite its limitations, the ability to use an in vitro model to study acute focal damage to cardiac tissue is a powerful tool to better understand the cellular processes occurring following acute cardiac injury. Although in vitro models of focal cardiac fibrosis exist (26–28), this model is uniquely able to capture the response to injury for cardiac cells (i.e., CMs and CFs) in surrounding regions. With this approach, we have high levels of control over the tissue architecture and composition and the damage, enabling the study of acute local injuries of different magnitudes and response of the remaining tissue. In the future, we propose that this model could be used to perturb functional interventions to reveal new targets that prevent the progressive deterioration of cardiac function seen in vivo. As engineered heart tissue models become more sophisticated, this model could be expanded to study other processes associated with the cardiac repair, such as immune cell activation or revascularization of injured tissues.

## SUPPLEMENTAL DATA

10.6084/m9.figshare.20020511 Supplemental Videos S1–S9: https://doi.org/10.6084/m9.figshare.20020511.

## GRANTS

S.L.D. acknowledges funding from National Science Foundation (NSF) Graduate Research Fellowship 1122374. This work was supported by NSF for Engineering Research Center for Cellular Metamaterials Grant EEC-1647837 (to S.L.D., B.P.S., E.L., and C.S.C.), NSF for Science and Technology Center for Engineering Mechanobiology Grant CMMI-1548571 (to C.S.C.), American Heart Association Career Development Award 856354 (to E.L.), and National Institute of Biomedical Imaging and Bioengineering Grant R21 EB028491 (to J.E.).

## DISCLOSURES

C.S.C. is a founder and own shares of Innolign Biomedical, a company that is developing engineered organ models for pharmaceutical research and development, and Satellite Biosciences, a company that is developing cell-based therapies. None of the other authors has any conflicts of interest, financial or otherwise, to disclose.

## AUTHOR CONTRIBUTIONS

S.L.D. and C.S.C. conceived and designed research; S.L.D. and B.P.S. performed experiments; S.L.D., B.P.S., and E.L. analyzed data; S.L.D., J.E., and C.S.C. interpreted results of experiments; S.L.D. prepared figures; S.L.D., J.E., and C.S.C. drafted manuscript; S.L.D., B.P.S., E.L., J.E., and C.S.C. edited and revised manuscript; S.L.D., B.P.S., E.L., J.E., and C.S.C. approved final version of manuscript.
